# Positive expressive writing interventions, subjective health and wellbeing in non-clinical populations: A systematic review

**DOI:** 10.1371/journal.pone.0308928

**Published:** 2025-05-21

**Authors:** Lauren M. Hoult, Mark A. Wetherell, Trudi Edginton, Michael A. Smith

**Affiliations:** 1 Department of Psychology, Northumbria University, Newcastle upon Tyne, United Kingdom; 2 Department of Psychology, City, University of London, London, United Kingdom; ECHS: Universidade de Tras-os-Montes e Alto Douro Escola de Ciencias Humanas e Sociais, PORTUGAL

## Abstract

Positive expressive writing has been increasingly researched over the past two decades due to its potential to serve as a low-intensity psychological self-help intervention. However, studies are heterogeneous in their methodologies and the health and wellbeing outcomes targeted, and it is unclear which outcomes are most reliably benefited by positive writing techniques. This systematic review aimed to determine the optimal conditions under which positive expressive writing interventions benefit subjective health and wellbeing in non-clinical populations. A systematic search was conducted across four databases (Web of Science, Scopus, PubMed, and ProQuest: APA PsychArticles) identifying peer-reviewed articles written in the English language from 1930 to August 2023. A total of 51 studies were identified and included seven different positive writing techniques: best possible self, positive experiences, gratitude, benefit finding, satisfaction processes, three good things and resource diary. Most consistent benefits were found for wellbeing and positive affect outcomes (e.g., optimism, happiness) whereas less consistent effects were reported for negative affect, psychological health (e.g., stress, anxiety) and physical health outcomes. Best possible self and gratitude interventions revealed most consistent benefits. Several moderators were identified indicating that benefits may depend on individual differences relating to wellbeing, emotional and social factors. While reasonably consistent benefits of positive expressive writing were observed for wellbeing outcomes, the quality of all studies included in the review was assessed to be poor or fair. Thus, it is clear that more rigorous methods, including intention-to-treat analyses and robust reporting of methods and findings are needed. Future work should also aim to replicate the moderation effects reported in the present review, to enable a better understanding of the individual differences which influence the efficacy of positive expressive writing effects.

## Introduction

Positive expressive writing is a therapeutic technique involving self-reflection and written emotional expression about specific positive topics (e.g., gratitude, positive experiences, best possible self) and associated thoughts, feelings and emotions. Such interventions developed as an adaption of the traditional written emotional disclosure (WED) paradigm; a technique which involves writing about a stressful or traumatic experience typically for 15–20 minutes over three to five consecutive days [1]. Considerable evidence demonstrates that WED leads to various psychological and physical health benefits in both clinical and non-clinical populations [2]. However, one potential drawback of traditional WED is that there are risks associated with completing the exercise without additional support due to the short-term negative emotions that often arise following negative disclosure. Therefore, researchers have cautioned against alone home-based application [3] which limits application of WED as a therapeutic technique. With positive writing, the possible risks discussed above are substantially minimised and it has the potential to serve as a self-administered low-intensity psychological intervention. Positive writing interventions align with positive psychology interventions (PPIs) which have been shown to enhance wellbeing and reduce or buffer against adverse health outcomes through promoting positive emotions, strengths and virtues [4]. Additionally, self-help techniques come with various benefits in that they are low-cost, accessible, and time-effective, and can be delivered to populations with time constraints and limited availability for individuals who need a psychological intervention (e.g., caregivers) [5,6].

Early positive writing studies followed a typical WED protocol, except the emotive content was altered to positive topics such as writing only about the benefits of a traumatic experience (benefit finding) [7] and one’s best possible self (BPS) [8]. Findings showed that these positive writing techniques led to similar health benefits as WED, but without immediate negative emotions. This spurred further research over the following two decades investigating other positive writing techniques with different protocols (e.g., writing duration, spacing) and targeting various health outcomes and populations. In order to ascertain the effectiveness of positive writing interventions, several reviews and meta-analyses have been conducted which either focus broadly on expressive writing interventions, or PPIs with varying methodologies. The largest and perhaps most influential meta-analysis on 146 experimental disclosure studies found small effects in improving a range of psychological and physical health outcomes in both clinical and non-clinical populations [2]. This review served as preliminary evidence to suggest that disclosing positive events is equally as beneficial as disclosing negative events, but without the associated short-term negative emotions. However, only a small number of positive writing studies were included in this review and the number of positive expressive writing studies have increased substantially since the review was published. In terms of PPIs, numerous systematic reviews with meta-analyses have been conducted which either focus broadly on all PPIs with varying definitions or the focus has been refined to one specific type of PPI (e.g., gratitude, best possible self) with varying modes of delivery (e.g., list writing, speaking, journaling). Several meta-analyses on PPIs with varying definitions have found consistent benefits in wellbeing, depression, anxiety, and stress in clinical and non-clinical populations, as well as in western and non-western societies [4,9–13]. Where meta-analyses and systematic reviews have focussed on single positive topics, gratitude interventions have been shown to bring about small improvements in psychological wellbeing including happiness, life satisfaction, and positive affect, as well as decreases in depressive symptoms [14,15], however findings on physical health and health behaviours are mixed [16,17]. Only one meta-analysis on PPIs has confined the review to a single positive topic and writing as the mode of disclosure. Carrillo and colleagues [18] conducted a review of the best possible self (BPS) intervention where individuals are asked to write about themselves in the future as if everything had gone as well as it possibly could. Findings showed that BPS interventions convey medium to moderate effects in enhancing wellbeing, optimism and positive affect compared to controls, as well as small effects for reducing negative affect and depressive symptoms, with some studies showing both increases in positive affect and decreases in negative affect.

At present, whereas other reviews have focused on expressive writing or PPIs more broadly, there are no existing reviews which have refined the scope to expressive writing as the mode of disclosure and focussed solely on positive topics. Existing positive writing studies are heterogeneous in their methodology (e.g., writing topic, protocol, comparison group), the specific health and wellbeing outcome variables targeted, and it is unclear which outcomes are most reliably benefited. These inconsistencies make it challenging to draw meaningful inferences regarding intervention effectiveness which is problematic if the technique it is to be tested and recommended as a psychological intervention. Therefore, this review aims to synthesise the literature investigating the effects of positive expressive writing interventions on self-reported health and wellbeing in non-clinical populations. This encompasses both subjective wellbeing (e.g., life satisfaction, positive affect, negative affect) [19] and psychological wellbeing (e.g., personal growth, purpose in life) [20], as well as self-reported psychological health (e.g., anxiety, depression, stress) and physical health. The focus is refined to subjective outcomes, as objective measures exceed the scope of the review in that they largely aim to elucidate the mechanisms underpinning the beneficial effects of positive expressive writing on health. Additionally, this review focuses on studies recruiting non-clinical populations, to avoid the potential confounds of having an existing physical or psychological health condition. The findings from this review will be able to inform the methodology and enhance the rigor of future positive expressive writing studies.

### Research questions

1) What are the optimal conditions under which positive expressive writing benefits self-reported physical and psychological health and wellbeing?
2) Which positive expressive writing techniques work best, on what health and wellbeing outcomes, and for whom?

## Method

### Protocol and registration

This systematic review was conducted according to the Preferred Reporting Items for Systematic Reviews and Meta Analyses (PRISMA) guidelines [21]. The protocol, eligibility criteria, data extraction, and quality assessment were preregistered on the Open Science Framework: https://osf.io/chwta.

### Search strategy

An initial systematic search was conducted across four electronic databases, including Web of Science (all databases), Scopus, PubMed, and ProQuest (APA PsychArticles), identifying articles published between 1930 (earliest available article on Scopus) and January 11th, 2021. Updated searches were conducted on 15th February 2022 and 9^th^ August 2023. Key search terms relating to positive writing topics, context of expression, writing, and self-reported health and wellbeing were included in systematic combinations with Boolean terms. Search terms were identified by cross-checking references from previous reviews and meta-analyses on expressive writing and positive psychology interventions, including positive writing studies [2,4]. The following search terms were used for all databases: (positiv* OR optimis* OR gratitude OR “three good things” OR “benefit finding” OR “post-traumatic growth” OR “best possible self” OR meaning* OR “life goals”) AND (express* OR emotion* OR reflect* OR reappraisal OR disclosure OR therapeutic OR therapy OR intervention OR exercise OR journal OR journaling OR diary OR letter) AND (writing OR written OR wrote OR write) AND (wellbeing OR well-being OR “physical health” OR “psychological health” OR “mental health” OR “physical symptoms” OR anxiety OR depression OR burnout OR stress OR distress OR “quality of life” OR satisfaction OR sleep). Searches were limited to peer-reviewed journal articles available in the English language. Relevant articles were hand searched.

### Eligibility criteria

#### Inclusion criteria (PICOS).

*Population*.   Non-clinical adult population (18^+^ years).
*Intervention.  S*elf-administered positive expressive writing intervention.

- Writing that aims to cultivate positive feelings, behaviours, or cognitions as opposed to writing that aims to reduce negative feelings, symptoms, problems, or disorders [4].
- Intervention that includes instructions to write expressively and in detail, as opposed to list writing, note taking, or ‘jotting down’ thoughts.
- Positive expressive writing must be the main intervention and not a component within a larger intervention programme comprising multiple therapeutic techniques.

*Comparison.*  Must have a control group (e.g., neutral writing control, non-writing control, active control, no-treatment control with pre- and post-measures).
*Outcomes.*   Subjective wellbeing, psychological wellbeing, psychological health, and physical health.
*Study design.*  Experimental design with random allocation of participants to two or more conditions.

#### Exclusion criteria.

Exclusion criteria encompassed: clinical populations (populations defined as clinical or currently receiving treatment for any psychological or medical conditions); participants under the age of 18, writing not based on a positive topic and was not expressive, positive writing not being the sole intervention, no control group, no subjective health and wellbeing outcomes (e.g., only including objective health and wellbeing outcomes or health behaviours), non-experimental designs; not a peer-reviewed article; not available in the English language; and no availability to full-text article.

### Selection process

The articles generated from each database were downloaded separately and combined into EndNote. Screening was completed in three stages (title, abstract, and full text). The initial search used a complete dual screening approach with two independent reviewers (LH and MS) who met to discuss discrepancies at each stage. Percentage of agreement was calculated, and inter-rater reliability was determined using Cohen’s kappa (κ). The updated searches were conducted by a single reviewer (LH) and final full text articles were checked by the second reviewer (MS) for discrepancies.

### Data extraction

Data extraction was conducted by a single reviewer (LH). This was based on previous meta-analytic reviews on experimental disclosure and modified for the purpose of a systematic review on positive expressive writing interventions. The following data were extracted from each study: report information (authors; year of publication), setting information (country; population), participant demographics (sample size; age; gender; ethnicity; education), type of positive writing intervention, type of control group, mode of disclosure (handwritten; typed), treatment information (number, duration, spacing and location of writing sessions), timing of post-test or follow-up, and methodological information (outcomes; moderators; attrition). Where studies included an intervention group additional to positive writing and did not report participant demographics or dropout per group, these characteristics reported were based on the total sample (such studies have been marked with an asterisk ‘*’). Sample size was based on the total number of participants included in the analysis in order to reflect the key findings reported. Attrition was based on dropout from randomisation to follow-up and is therefore not a representation of percentage dropout from reported sample size. Ethnicity and education characteristics are based on the majority within the sample. Studies including a writing control group were labelled as ‘neutral writing’. Where studies have not explicitly stated that typing, handwriting, or pen-and-paper methods were used, no mode of disclosure has been recorded.

### Quality assessment

Each article was quality assessed by a single reviewer (LH) using the National Institutes of Health (NIH) Quality Assessment of Controlled Intervention Studies: https://www.nhlbi.nih.gov/health-topics/study-quality-assessment-tools. The tool comprises 14 criteria assessing description and adequacy of randomisation, concealment of treatment allocation, blinding of participants, providers, and people assessing outcomes, similarity of groups at baseline, overall and differential group dropout, adherence to the intervention protocols, similarity of background treatments, validity and reliability of outcome assessment, statement of power calculation, prespecified outcomes, and analysis of all randomised participants (i.e., intention-to-treat). The overall study quality was determined by assessing the internal validity of each study based on the criterion. Studies with low risk of bias were rated as good, studies with some concerns for bias were rated as fair and studies with high risk of bias were rated as poor. Studies with fatal flaws (e.g., high overall (>20%) and/or differential dropout rates (≥15%), completers only analysis) were considered significant risk for bias and were rated as poor.

## Results

### Study selection

**Fig 1:**
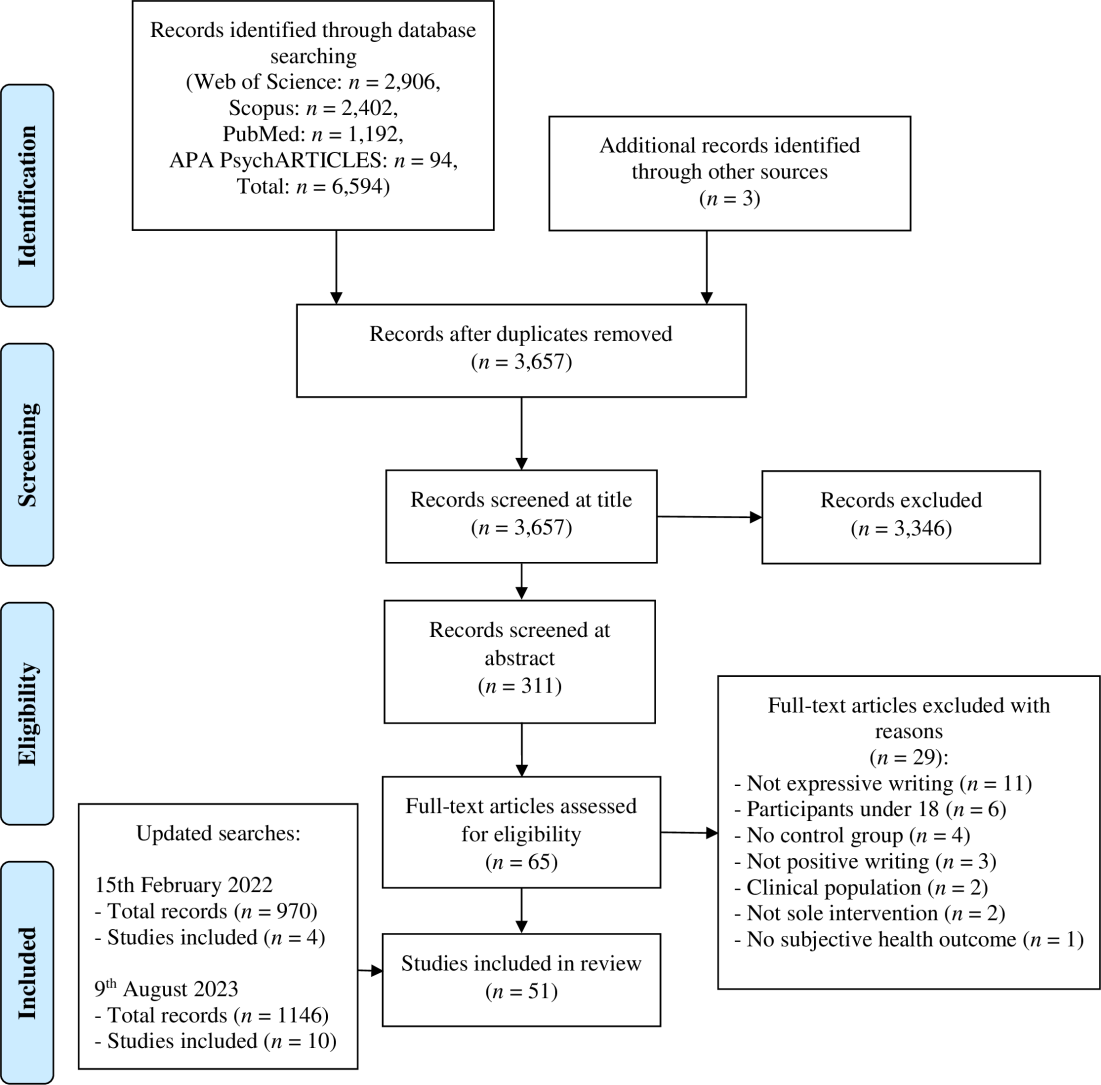
PRISMA flow diagram of screening and selection process.

A total of 6,594 articles resulted from the four databases and a further three articles were identified through hand searching relevant articles (Fig 1). Duplicates were removed prior to screening on Endnote using the ‘Find Duplicates’ function (*n* = 2,940) and additional duplicates identified during the title screening were deleted manually (*n* = 6). Initial screening of article titles (*n* = 3,657) resulted in the exclusion of 3,346 articles (96%, κ = .71). Secondary screening of article abstracts (n = 311) resulted in the exclusion of 246 articles (89%, κ = .70). Remaining articles (*n* = 65) were screened at full text and a further 29 articles were removed (92%, κ = .83). From the initial search, a total of 36 articles were remaining, and one article consisted of two eligible studies, therefore 37 studies were considered for review. The 15^th^ February 2022 updated search produced 970 articles and duplicates were removed (*n* = 449). Articles were screened according to the aforementioned eligibility criteria at title (*n* = 521), abstract (*n* = 115) and full text (*n* = 26). Four additional articles were considered eligible for review. The 9^th^ August 2023 updated search produced 1146 articles and duplicates were removed (*n* = 376). Articles were screened at title (*n* = 770), abstract (*n* = 123) and full text (*n* = 43). Nine articles were considered eligible for review and one additional article was found through hand searching. Therefore 51 studies were included in the review (Table 1).

**Table 1:** Characteristics and key findings of final studies included in the review.

First author (year), county	Sample (% attrition)	Group treatment *Moderators*	Timing of health outcomes	Key findings
Allen et al. (2020) UK	Adults with high negative affectivity, 98.6% UK residents *N *= 72 (52%) *M*_*age*_* *= 28.5 ± 8.7 Female: 86.1%	I: IPE C: NW_a_ Typed in quiet location of choice 3 x 20 mins on consecutive days *Social inhibition*	Immediately post-writing: state anxiety 1 month: depression, general anxiety, perceived stress, perceived stress reactivity, physical symptoms	High social inhibition was associated with greater reductions in depression and perceived stress reactivity in IPE relative to control NS main effects or interactions on all other outcomes
*Antal et al. (2005) USA	Undergraduate psychology students^a^ *N *= 85 (16.7%) Female: 84.7%	I: PE C: NW_t_ Typed (56.5%) or handwritten (43.5%) in private room 4 x 20 mins on consecutive days	Immediately pre-final writing and 1 month: state anxiety, depression, suicidal ideation 2 months: health centre visits	Main effects of time where reports of depression and suicidal ideation were lower at pre-final writing and follow-up relative to pre-test, and reports of state anxiety were lower at pre-final writing relative to pre-test and follow-up NS effects on health centre visits NS interactions on all outcomes
Ashley et al. (2011) UK	Informal caregivers *N *= 99 (33.1%) *M*_*age*_* *= 54.9 ± 11.62 Female: 85.9% White: 87.9% University degree: 32.3%	I: IPE C: NW_l_ Handwritten at home or quiet location of choice 3 x 20 mins on consecutive days *Alexithymia*	2 weeks, 2 and 6 months: depression, general anxiety	Low alexithymia was associated with decreased depression and/or anxiety at all follow-ups in IPE, and this was relative to control for depression at 2 weeks and 2 months NS main effects on all outcomes
*Austenfeld et al. (2006) USA	Third-year medical university students^a^ *N *= 42 (11.1%) *M*_*age*_* *= 26.41 ± 4.04 Male: 55% White: 84%	I: BPS (medical career) C: NW_t_ Handwritten in private room 3 x 25 mins at least 1-week apart within 8 weeks *Emotional processing coping (EP), emotional expression coping (EE)*	3 months: depression, negative mood (hostility, sadness, fear, guilt), health centre visits, physical symptoms	Low EP and low EE were associated with lower depression at follow-up in BPS relative to control Low EP was associated with fewer health centre visits in BPS relative to control NS main effects or interactions on all other outcomes
*Austenfeld et al. (2008) USA	Undergraduate psychology students^a^ *N *= 63 *M*_*age*_* *= 19 Female: 69.8%	I: BPS C: NW_t_ 3 x 20 mins at weekly intervals *Emotional processing coping (EP), emotional expression coping (EE)*	1 month: depression, hostility, physical symptoms, health centre visits	Low EP was associated with fewer heath centre visits and high EP was associated with more health centre visits in BPS relative to control Low EE was associated with lower hostility and high EE was associated with higher hostility in BPS NS main effects or interactions on all other outcomes
Auyeung et al. (2019) China	Chinese university students *N *= 100 (28.1%) *M*_*age*_* *= 22.82 ± 3.35 Female: 73%	I: BPS (various domains of self) C: List 5 events and describe one in detail Typed 6 x 10–15 mins on consecutive days	1-day primary outcomes: depression, flourishing; secondary outcomes: positive affect, need satisfaction	Depression, flourishing, positive affect and autonomy subscale of need satisfaction increased in BPS relative to control NS main effects and interactions on relatedness and competence subscales of need satisfaction
Basten-Gunther et al. (2022) Germany	German adults *N* = 40 *M*_*age*_* *= 39.9 ± 13.5 Female: 39.9%	I: BPS (+ post-visualisation) C: NW_t_ Handwritten in lab 1 x 15mins (+ 5min) *Dispositional pain catastrophising (DPC)* *Dispositional optimism*	Immediately post-writing: situational pain catastrophising (SPC), state optimism	In the BPS condition, low DCP was associated with greater reductions in SPC, whereas high DPC was associated with increases in SPC NS main effects of condition on state optimism NS moderation of dispositional optimism
Bhullar et al. (2011) Australia	General population *N *= 90 (21.7%) *M*_*age*_* *= 31.98 ± 9.94 Female: 83.5%	I: Satisfaction processes C: NW_a_ Handwritten in location of choice 3 x 20 mins on consecutive days	2 weeks: life satisfaction, positive affect, psychological wellbeing, social wellbeing, depression, anxiety, stress, general physical health	Life satisfaction, positive affect, psychological wellbeing and social wellbeing increased, and depression, anxiety and stress decreased in satisfaction processes writing relative to control NS effect on general physical health
*Boselie et al. (2023) Netherlands	Healthy adults^a^ *N* = 141 (39.5%) *M*_*age*_* *= 22.1 ± 7.8 Female: 84.4%	I: BPS x 2 (writing, writing + post-visualisation) C: NW_t_ Typed 1 x 15 (+ 5mins)	Immediately post-treatment: positive affect, negative affect, optimism	Both BPS conditions showed improved optimism (increased positive future expectancies and decreased negative future expectancies) and affect (increase positive affect and decreased negative affect) relative to control
Burton et al. (2004) USA	Undergraduate psychology students *N *= 90 *M*_*age*_* *= 18.58 ± .95 Female: 73.3% European American: 85%	I: IPE C: NW_a_ Handwritten in lab 3 x 20 mins on consecutive days	Immediately post-writing: positive affect, negative affect 3 months: health centre visits	Positive affect increased in IPE relative to control Number of health centre visits were lower in IPE relative to control (buffering effect rather than reduction) NS effect on negative affect
*Burton et al. (2008) USA	Undergraduate psychology students^a^ *N *= 49 Female: 73.5% Caucasian: 88%	I: IPE C: NW_a_ Typed in private room 2 x 2 mins on consecutive days	Immediately post-writing: Positive affect, negative affect 4-6 weeks: physical symptoms	Physical symptoms were lower in IPE relative to control NS effect on positive affect and negative affect
Burton et al. (2009) USA	Undergraduate psychology students *N *= 38 *M*_*age*_* *= 19.16 ± 1.7 Female: 71.1% Anglo American: 79%	I: IPE C: NW_a_ Typed in private room 3 x 20 mins on consecutive days	Immediately post-writing: Positive affect, negative affect 4-6 weeks: physical symptoms	Positive affect increased, negative affect decreased, and physical symptoms were lower in IPE relative to control
Carrillo et al. (2020); *Study 1* Spain	General population (mostly university students) *N *= 112 (21.4%), *M*_*age*_* *= 21.76 ± 3.63 Female: 76.8%	I: BPS x 3 (past, present, future orientated) C: NW_t_ Typed in lab (day 1) and home (days 2–7) 7 x 15 mins on consecutive days	Immediately post-treatment primary outcomes: positive affect, negative affect; secondary outcomes: life satisfaction, happiness, optimism, self-efficacy, self-satisfaction	Main effect of time where all outcomes improved NS interactions on all outcomes
Carrillo et al. (2020); *Study 2* Spain	General population *N *= 107 (21.5%), *M*_*age*_* *= 23.86 ± 6.25 Female: 82.2%	I: BPS x 3 (past, present, future orientated) C: NW_t_ Typed at home 7 x 15 mins on consecutive days	Immediately post-treatment primary outcomes (single item scales): Positive affect, negative affect; secondary outcomes: life satisfaction, optimism, self-efficacy	Main effect of time where all outcomes improved NS interactions on outcomes
Contractor et al. (2022) USA	Undergraduate psychology students *N *= 43 (13.2%) *M*_*age*_* *= 22.51 ± 4.55 Female: 83.7% White: 74.4%	I: PE (present tense with sensory details) C: Semantic fluency task Typed 2 x 30 mins ~1-week apart	Immediately post-treatment: Affect, posttraumatic cognitions	NS effects on all outcomes
Duan et al. (2021) USA	Undergraduate educational technology students *N *= 90 (26.7%) *M*_*age*_* *= 19.14 ± 0.89 Female: 69%	I: BPS (work-related) C: NW_t_ Typed in lab in groups 3 x 15 minutes at weekly intervals	Immediately post-treatment, 1 and 2 months: Flourishing, subjective wellbeing	Subjective wellbeing increased in control relative to BPS at 1-month follow-up NS effects on flourishing
*Fekete et al. (2022) USA	American adults^a^ *N* = 54 (53.6%) *M*_*age*_* *= 40.86 ± 17.4 Female: 86.1% White: 93.7% College degree or higher: 84.8%	I: Gratitude (life) C: No treatment Typed online 7 x 5–10 mins daily	Immediately post-treatment and 1-month: state gratitude, stress, anxiety, depression positive affect, negative affect, physical symptoms	State gratitude increased from baseline to one week, but this was not sustained at one month in gratitude condition Stress and negative affect decreased from baseline to one week and one month in gratitude relative to control NS effects on anxiety, depression, positive affect and physical symptoms
*Frein et al. (2014) *Study 1* USA	Undergraduate psychology students^a^ *N *= 39 *M*_*age*_* *= 20.6 Female: 7.7%	I: BPS x 2 (future self, loved one) C: NW_a_ 4 x 15 minutes on consecutive days	Immediately post-writing: positive affect, negative affect	Positive affect increased in BPFS relative to BPFL and control Positive affect increased in BPFL relative to control NS effect on negative affect
Fuju et al. (2022) Japan	Family caregivers *N* = 22 (15.4%) *M*_*age*_* *= 62.6 ± 11.05 Female: 77.27%	I: Three good things C: Food diary Daily for 4 weeks	Immediately post-treatment primary outcomes: caregiver distress, depression; secondary outcomes: quality of life, caregiver burden, positive cognitive appraisal, positive feelings	Depression and positive cognitive appraisal improved in three good things condition relative to control NS differences on all other outcomes
Gallagher et al. (2020) Ireland	Informal caregivers *N *= 88 (48.9%) *M*_*age*_* *= 47.87 ± 8.93 Female: 94.3%, White Irish: 89.8% University degree: 39.8%	I: BF (caregiving) C: NW_w_ Handwritten 6 x 3 per week, 3–4 sentences minimum	Immediately post-treatment, 3 months: benefit finding, caregiver quality of life, depression, general anxiety	NS effects and interactions on all outcomes
*Guastella et al. (2008) Australia	Undergraduate psychology students^a^ *N *= 79 (6.6%) *M*_*age*_* *= 24.08 ± 7.75 Female: 68.8%	I: BF (upsetting experience) C: NW_e_ Handwritten in quiet location of choice 3 at weekly intervals	2-months: depression, general anxiety, post-traumatic growth, subjective stress	Post-traumatic growth increased in BF relative to control NS effects or interactions on all other outcomes
Hansen et al. (2021) UK	General population resident in UK *N* = 91 (41.9%) *M*_*age*_ = 39.13 ± 14.35 Female: 63.7%	I: BF (Covid-19 pandemic) C: NW_t_ Typed 3 x 15 mins on consecutive days *Perseverative thinking*	Immediately post-writing: state anxiety 2 weeks: depression, general anxiety, perceived stress, physical symptoms	State anxiety decreased to a greater extent in BF relative to control Main effect of time where anxiety and depression decreased NS moderation
Heekerens et al. (2020) Germany	Undergraduate psychology students *N *= 188 (8.3%) *M*_*age*_* *= 22.35 ± 5.04 Female: 78.7%	I: BPS + post-visualisation C: NW_t_ Handwritten 4 x 20 mins on separate days within 1 week	Immediately post-treatment, 1-week: Positive affect, negative affect, life satisfaction, hope, future expectations, gratitude	Positive affect increased at both follow ups in BPS relative to control NS effects on positive future expectations, life satisfaction, gratitude and hope
*Heekerens et al. (2022) Germany	German adults^a^ *N *= 321 *M*_*age*_* *= 43.26 ± 12.67 Female: 57.2%	I: BPS, gratitude letter C: NW_t_ Typed 1 x 15 mins *Emotional self-awareness (BPS)* *Trait gratitude (gratitude letter)*	Post-treatment: optimism, gratitude, positive affect	Gratitude increased in gratitude condition relative to control Optimism increased in BPS conditions relative to control NS effects on positive affect for both interventions NS moderation
*King et al. (2000) USA	Undergraduate psychology students^a^ *N* = 55 *M*_*age*_* *= 20.95 ± 3.42 Female: 70.3%	I: BF (traumatic experience) C: NW_a_ Handwritten in private lab 3 x 20 mins on consecutive days	Immediately post-writing: positive affect, negative affect 3 and 5 months: health centre visits	Health centre visits were fewer in BF relative to control at both follow-ups NS differences in affect
*King (2001) USA	Undergraduate psychology students^a^ *N *= 81 *M*_*age*_* *= 21.04 ± 3.15 Female: 85.2% European American: 87%	I: BPS C: NW_t_ Handwritten in private room 4 x 20 mins on consecutive days	Immediately post-writing: mood 3 weeks: psychological wellbeing 5 months: health centre visits	Positive affect and psychological wellbeing increased in BPS relative to control Health centre visits were lower in BPS relative to control
*Kloss et al. (2002) USA	Undergraduate psychology students^a^ *N *= 129 (3.7%) *M*_*age*_* *= 18.80 Female: 51.2%	I: Happiest experiences C: NW_t_ Handwritten in private room 3 x 20 mins on consecutive days	Immediately post-writing: state anxiety ~9 weeks: trait anxiety, depression, physical symptoms, health centre visits, illnesses, sick days	Main effect of time where trait anxiety decreased NS effects on state anxiety, physical symptoms, health centre visits and self-report of illness NR depression outcome
Kupeli et al. (2018) UK	Undergraduate psychology students *N *= 57 (23%) *M*_*age*_* *= 20.38 ± 4.04 All female British: 44% A Level: 67%	I: IPE C: NW_r_ Handwritten at home 3 x 15 mins on consecutive days	2-months primary outcome: disordered eating; secondary outcomes: perceived stress, mood, self-criticism/self-reassurance	Dietary restraint subscale of disordered eating decreased in IPE relative to control NS effects on all other outcomes
*Layous et al. (2013) USA	Undergraduate psychology students^a^ *N *= 119 (9.2%) *M*_*age*_* *= 19.10 ± 1.77 Female: 71.8% Asian American: 30%	I: BPS x 4 (peer vs no peer testimonial, in-person vs online) C: NW x 2 (list writing, describe one in detail, control vs in-person) Handwritten and typed 4 x 15 minutes weekly	Immediately post-treatment: positive affect, need satisfaction (relatedness, autonomy, competence), flow	Increased positive affect and flow in BPS relative to control BPS + peer testimonial increased positive and flow relative BPS + no testimonial Both BPS + testimonial and no testimonial online showed increases in PA, flow and relatedness relative to control NS effects on need satisfaction, autonomy and competence
Layous et al. (2017) *Study 3* USA & South Korea	University students *N *= 291 (13.8%) *M*_*age*_* *= 20.8 ± 2.49 Female: 59.5% Asian: 43.4%	1: Gratitude (life) C: NW_t_ Typed online 1 x 8mins	Immediately post-treatment: positive emotion, negative emotion, state gratitude, connectedness	Increased positive emotions and decreased negative emotions following gratitude relative to control NS differences in connectedness
Lovell et al. (2016) UK	Caregivers of children with autism *N *= 33 (10.8%) *M*_*age*_* *= 44.56 ± 4.68 Female: 84.9% University degree: 63.6%	I: BF (caregiving) C: NW_l_ Handwritten at home 3 x 20 minutes on consecutive days	1, 3 months: depression, general anxiety, perceived stress	Anxiety was less likely to be in clinical range in BF at 3-months relative to control (indicating a buffering effect rather than reduction) NS effects or interactions on all other outcomes
*Marlo et al. (1999) USA	Undergraduate psychology students^a^ *N *= 100 (10%) Aged 19–21 = 58% Female: 66% Caucasian: 75%	I: IPE C: NW_a_ Handwritten in groups 4 x 20 minutes, twice per week and two days apart	Immediately post-writing: physical sensations, state anxiety 1-month: psychological health, physical symptoms	Main effect of time where psychological health improved Physical sensations increased in IPE relative to control NS effects on state anxiety and physical health
Moieni et al. (2019) USA	Middle aged women between 35–50 *N *= 68 (10.5%), *M*_*age*_* *= 42.6 ± 4.8 All female White: 70.6%	I: Gratitude letter (various topics) C: NW_a_ Location of choice 6 x 5–10 mins minimum at weekly intervals *Psychological distress*	Immediately post-treatment: state gratitude, trait gratitude	State gratitude increased in gratitude relative to control NS effects on trait gratitude NS moderation
*Nagurney (2013) USA	Undergraduate psychology students^a^ *N *= 276 *M*_*age*_* *= 20.81 ± 3.99 Female: 82.6% Caucasian: 61.9%	I: Happiest relationship C: NW_t_ Handwritten at home 3 x 20 minutes on consecutive days	1-week from baseline: depression, general anxiety, positive affect, negative affect	NS effects on all outcomes
Nelson et al. (2010) USA	Undergraduate psychology students *N *= 118 Female: 65.3%	I: Past peak experience coping with a challenge C: NW_t_ Handwritten in groups 1 writing session	Immediately post-writing: Positive affect, negative affect, stress appraisal, optimism, test anxiety	Positive affect and optimism increased, negative affect and test anxiety decreased, and stress was appraised more favourably in IPE relative to control
*Regan et al. (2023) USA	Australian adults^a^ *N *= 470 (63.2%) *M*_*age*_* *= 47.8 ± 16.9 Female: 52.2% White: 87%	I: Gratitude letter x 2 (social, non-social) C: NW_a_ Typed online 7 sessions on consecutive days	Immediately post-treatment and 1 week: gratitude, life satisfaction, positive affect, negative affect, indebtedness, need satisfaction, elevation	Following the social gratitude, indebtedness, elevation, gratitude, positive affect, and connectedness increased at post-test relative to control Following non-social gratitude, gratitude increased at post-test relative to control
Renner et al. (2014) The Netherlands	Undergraduate university students *N *= 40 (80%) *M*_*age*_* *= 22.1 ± 3.87	I: BPS (1 min thinking, 15 mins writing, 5 mins mental imagery) C: NW_t_ In a laboratory 1 x 15 minutes	Immediately post-treatment: positive affect, negative affect, mood (positive-negative, glad-dull, secure-anxious, happy-sad)	Positive affect and positive, glad and happy subscales of mood increased in BPS relative to control NS differences in other outcomes
Round et al. (2022) UK	Teachers and non-teacher employees *N *= 66 (36.5%) *M*_*age*_* *= 38.1 ± 12.3 Female: 81.8%	I: PE C: NW_a_ Typed 3 x 20 minutes on consecutive days	Immediately post-writing: state anxiety 4 weeks: burnout, job-satisfaction, trait anxiety, perceived stress, physical symptoms	State anxiety decreased and satisfaction with contingent reward increased in PE relative to controls for both teachers and non-teachers Perceptions of promotion likelihood increased in PE relative to control in non-teachers, but not teachers NS effects on trait anxiety, perceived stress and physical symptoms
*Shapira et al. (2010) Canada	Canadian adults *N *= 125 (79.7%)^a^ *M*_*age*_* *= 34 Female: 81.5% Caucasian: 79.4%	I: Optimistic future C: NW_m_ Typed 7 x 5–15 minutes on consecutive days	Immediately post-treatment, 1, 3, and 6 months: depression, happiness	Depression decreased at 1 month and 3 months in optimism relative to control Happiness increased immediately post-treatment and at 3- and 6-month follow-ups in optimism relative to control
Shin et al. (2020) USA	American undergraduate university students *N *= 581 (7.8%) *M*_*age*_* *= 20.25 ± 1.47 Female: 79.6% White: 52.7% Asian: 47.3%	I: Gratitude letter (to parents) C: NW_f_ Typed in location of choice 1 x 20 minutes *Race, familial collectivism, parent-child relationship*	2-weeks: positive affect, negative affect, generic gratitude, gratitude towards parents	Positive affect decreased in control relative to gratitude writing which remained stable Low parent-child relationship quality was associated with higher positive affect in gratitude Low quality of parent-child relationship and high familial collectivism was associated with increased generic gratitude and positive affect in gratitude NS moderation of race
Smith et al. (2018) UK	Heathy adults *N *= 69 (2.8%) *M*_*age*_* *= 28.2 ± 12.4 Female: 73.2%	I: IPE C: NW_a_ Handwritten in quiet choice of location 3 x 20 mins for consecutive days *Type D personality*	Immediately post-writing: state anxiety 1-month: trait anxiety, perceived stress, physical symptoms	State anxiety, trait anxiety and perceived stress decreased in IPE relative to control NS effects on physical symptoms NS interactions or moderation effects
Teismann et al. (2014) Germany	German adults *N *= 64 *M*_*age*_* *= 29.1 ± 8.42 Female: 62.5%	I: Life goals C: NW_a_ Handwritten in private room 3 x 20 mins for consecutive days	Immediately post-writing: positive affect Immediately post-treatment: positive affect, ruminative thinking	Ruminative thinking decreased from pre- to post-treatment Positive mood decreased immediately after first and third writing sessions in life goals NS group differences in outcomes from pre- to post-treatment
*Timmons et al. (2018) USA	Mothers of children with ASD under age 18^a^ *N *= 64 (22%), *M*_*age*_* *= 39.70 ± 6.92 All female White: 73.4% College degree: 65.6%	I: Gratitude letter x 2 (general, child-specific) C: NW (list writing) Typed 8 x ~ 15 minutes at ~ 1-week intervals	Within 1-week post-treatment, 1 month: benefit finding, depression, happiness, life satisfaction, parenting cognitions (satisfaction, self-efficacy), optimism	Main effect of time where depression, happiness, life satisfaction, parenting self-efficacy, optimism and benefit finding improved Parenting satisfaction increased for child-specific gratitude letter and control conditions, but this change was not evident in generic gratitude letter
Toepfer et al. (2009) USA	University students *N *= 84 *M*_*age*_* *= 26.7 ± 8.44 Female: 85% Caucasian: 77%	I: Gratitude letter (recipient not specified) C: No treatment Handwritten or typed 3 sessions at ~ 2-week intervals	Immediately post-writing: gratitude, life satisfaction, happiness	Happiness increased to a greater extent in in gratitude writing relative to control Gratitude increased in gratitude writing relative to a decrease in control Main effect of time where life satisfaction increased for both conditions
Toepfer et al. (2012) USA	University students *N *= 219 *M*_*age*_* *= 25.7 ± 11 Female: 85.8% Caucasian: 89%	I: Gratitude letter (person of choice) C: No treatment Handwritten or typed 3 sessions at weekly intervals (79% took 15–30 min)	Within 24 hours post-treatment: gratitude, life satisfaction, happiness, depression	Happiness, life satisfaction and depression improved in gratitude relative to control NS differences in gratitude
*Toepfer et al. (2016) Germany	Undergraduate psychology students^a^ *N *= 195 *M*_*age*_* *= 23.41 ± 3.16 Female: 84%	I: Resource diary C: No treatment Handwritten 3 per week on consecutive days at weekly intervals for 4 weeks *Baseline wellbeing, brooding*	Immediately post-treatment: resource realisation	Moderation whereby lower baseline wellbeing was associated with increased coping with daily hassles, social support and commitment in IPE relative to control Moderation whereby lower brooding was associated with higher wellbeing and self-esteem in IPE relative to control
Troop et al. (2013) UK	University students *N *= 46 *M*_*age*_* *= 25.8 ± 9.3 Female: 67.4%	I: Life goals C: NW_r_ Handwritten in groups 3 x 15 mins within 1 hour with 5-min breaks	2-weeks: Self-criticism and dependency, stress, positive affect	Self-criticism decreased in life goals relative to control (no change) Self-reassurance decreased in control relative to life goals (no change) NS differences in stress or positive affect
Walsh et al. (2022) USA	French-speaking employees *N *= 224 *M*_*age*_* *= 37.17 ± 9.08 Female: 71.4% College degree: 46.6%	I: Gratitude letter x 3 (kindness, health, work) C: List writing Typed 1 x 8 mins	Immediately post-treatment: state gratitude, connectedness, elevation, humility, negative affect, indebtedness, guilt, embarrassment, discomfort, shame, improvement motivation	Gratitude, elevation, indebtedness and guilt improved in kindness gratitude relative to control Elevation, indebtedness, guilt and discomfort improved in health gratitude relative to control Elevation, humility and indebtedness improved in work gratitude relative to control NS differences in connectedness, negative affect, shame and improvement motivation
Walsh et al. (2023) USA	University students *N *= 916 (17.1%) *M*_*age*_* *= 19.4 ± 2.1 Female: 67.7% Asian: 42.4%	I: Gratitude letter x 3 (private, text, social media) C: NW_t_ 1 session	Immediately pre-writing: gratitude, positive emotions, negative emotions, social emotions, life satisfaction, elevation, connectedness, loneliness	All gratitude conditions showed increased gratitude, positive emotions, life satisfaction, elevation, connectedness and support, as well as decreased loneliness relative to control NS differences in social emotions
*Wing et al. (2006) Australia	Australian adults^a^ *N *= 164 (6.9%) *M*_*age*_* *= 40.30 ± 16.04 Female: 64%	I: IPE x 2 (one with emotion regulation cue) C: NW_t_ Handwritten in quiet location of choice 3 x 20 mins on consecutive days	Immediately post-treatment, 2-weeks: Life satisfaction	Life satisfaction increased at post-treatment and follow-up in IPE + EMO NS differences between conditions at post-treatment or follow-up
Wong et al. (2009) USA	Undergraduate educational psychology students *N *= 157 (3.7%), *M*_*age*_* *= 21.64 ± 3.96 All male White: 54.5%	I: BPS (connectedness with romantic partner) C: NW_h_ Typed in groups in lab 3 x 20 mins within 1 week	Immediately post- treatment; 4-weeks: Restrictive emotionality, personal growth, psychological distress	Psychological distress decreased from pre-treatment to follow-up, but not post-treatment to follow-up in BPS relative to control NS differences in other outcomes

*I, intervention group; C, control group; NS, non-significant; NR, not reported; IPE, intensely positive experiences; PE, positive experiences; BPS, best possible self; BF, benefit finding; NW, neutral writing; NW*_*a*_*, aspects of daily life; NW*_*t*_*, time management: NW*_*f*_*, facts about parents; NW*_*e,*_ *environments; NW*_*r*_*, review a book or film; NW*_*m*_*, early memory; NW*_*h*_*, facts about human relationships; immediately post-writing, after each writing session; immediately post treatment, after the entire treatment.*

^a^ *demographics and attrition represent total sample for groups not reported here*

### Study characteristics

#### Participant characteristics.

Studies were mostly conducted in the USA (*n *= 26) followed by the UK (*n *= 8), Germany (*n *= 5), Australia (*n *= 3), The Netherlands (*n* = 2), Spain (*n *= 2), Canada (*n *= 1), China (*n *= 1), Ireland (*n *= 1) and Japan (*n* = 1). One study was conducted across both the USA and South Korea. Twenty-eight studies recruited participants from a university student population of which 18 were from a psychology cohort. Other studies recruited from the general population (*n* = 14), caregivers (*n *= 5), full-time employees (*n* = 2), adults with high negative affectivity (*n* = 1) and middle-aged women (*n* = 1). Recruited sample sizes ranged from 26 to 1105 participants, however sample sizes included in subsequent analyses ranged from 22 to 958 participants (*M *= 160). Thirty-four studies had less than 20% attrition and on average dropout was relatively moderate (16%) for all studies included in the review. The average age of participants ranged from 18.48 to 62.60 years old (*M*_*age*_ = 29.12, *SD*_*age *_= 6.92) and most studies (*n* = 32) included a high percentage of females (>70%). Of the 21 studies that reported ethnicity, the majority had a higher number of White/Caucasian participants (*n* = 14). In addition to the 28 studies that recruited samples from a university population, four studies also reported that the majority of participants had a college or university degree.

#### Intervention characteristics.

Six types of positive expressive writing interventions were identified in the review which included writing about ones best possible self (*n *= 18), positive experiences (*n* = 15), a gratitude letter (*n* = 11), benefit finding (*n* = 5), satisfaction processes (*n* = 1), three good things (*n* = 1) and a resource diary (*n* = 1). Writing objectively about various neutral or non-emotive topics were the most commonly used control condition and included writing about time management (*n* = 20), various aspects of daily life (*n* = 13), neutral-coloured landscape pictures (*n* = 2), a review of a book or film (*n* = 2), an early memory (*n* = 1), neutral environments (*n* = 1), objective facts about parents (*n* = 1), facts about human relationships (*n* = 1), and the weather (*n* = 1). Studies adopting other types of controls included no treatment (*n* = 4), list writing (*n* = 3), keeping a food diary (*n* = 1) and completing a sematic fluency task (*n* = 1). Where mode of disclosure and location of writing were reported, writing was handwritten (*n* = 22), typed (*n* = 20), handwritten or typed (*n* = 4) and either in a private room (*n* = 9), location of choice (*n* = 9), home (*n* = 7), in groups (*n* = 5) or in a laboratory (*n* = 3). Intervention duration in total minutes ranged from two 2-minute writing sessions to seven 15-minute writing sessions. Spacing of writing sessions ranged from 3 within 1 hour to weekly intervals. However, the most commonly used treatment followed the traditional written disclosure protocol of 3–4 writing sessions completed for 15–20 minutes over consecutive days (*n* = 19). Seventeen studies assessed outcomes immediately pre- and/or post-each writing session. Follow-ups ranged from immediately post-treatment to six months and the majority of studies included post-test or follow-up within a one-month period (*n* = 36). Outcomes were heterogenous and included various measures of psychological and physical health and wellbeing. Fifteen potential moderators were identified.

### Risk of bias

Table 2 summarises quality ratings for each study. The majority of studies (*n* = 32) were rated as poor and this was primarily due to fatal flaws of completers only analysis (*n* = 24), high overall dropout (*n* = 6), quasi-experimental design (*n* = 1), and lacking adherence to the intervention protocol (*n* = 1). It is worth noting that of the 23 studies that analysed all randomised participants, 15 had no attrition and only seven used an appropriate analysis (e.g., intention-to-treat) following dropout. The majority of remaining studies were rated as fair due to allocation bias risk as a result of underreporting participant randomisation and blinding (*n* = 17). An additional two studies were rated as fair for being statistically underpowered and having risk of confounders.

**Table 2:** Quality assessment ratings of final articles.

	1. Described as randomised or an RCT?	2. Adequate randomisation?	3. Concealed treatment allocation?	4. Blinding of participants and providers?	5. Blinding of people assessing outcomes?	6. Similarity of groups at baseline?	7.Overall dropout ≤20% from randomisation?	8. Differential group dropout ≤15% points?	9. High adherence to intervention protocols?	10. Similar background treatments?	11. Valid and reliable measures?	12. Reporting of sufficient sample size?	13. Prespecified outcomes and analyses?	14. Analysis of all randomised participants?	Quality rating
Allen et al. (2020)	Y	?	?	N	?	Y	N	Y	Y	Y	Y	Y	Y	N	Poor
Antal et al. (2005)	Y	?	?	N	?	Y	Y	?	Y	Y	Y	?	N	N	Poor
Ashley et al. (2011)	Y	Y	N	N	?	Y	N	Y	Y	Y	Y	Y	N	N	Poor
Austenfeld et al. (2006)	Y	?	?	N	?	Y	Y	?	Y	Y	Y	?	N	N	Poor
Austenfeld et al. (2008)	Y	?	Y	Y	?	Y	Y	Y	Y	Y	Y	?	N	Y	Fair
Auyeung et al. (2019)	Y	Y	Y	?	?	Y	N	Y	N	Y	Y	Y	N	Y	Fair
Basten-Gunther et al. (2022)	Y	?	N	?	?	Y	Y	Y	Y	Y	Y	N	N	Y	Fair
Boselie et al. (2023)	Y	?	?	?	?	Y	N	?	Y	Y	Y	Y	N	N	Poor
Bullar et al. (2011)	Y	?	?	?	?	Y	N	N	Y	Y	Y	?	N	N	Fair
Burton et al. (2004)	Y	?	?	?	?	?	Y	Y	Y	Y	Y	?	N	Y	Fair
Burton et al. (2008)	Y	?	?	?	?	Y	Y	Y	Y	Y	Y	?	N	Y	Fair
Burton et al. (2009)	Y	?	?	?	?	?	Y	Y	?	Y	Y	?	N	Y	Fair
Carrillo et al. (2020); S1	Y	Y	Y	N	?	Y	N	Y	?	Y	Y	Y	N	Y	Poor
Carrillo et al. (2020); S2	Y	Y	Y	N	?	Y	N	Y	?	Y	Y	Y	N	Y	Poor
Contractor et al. (2021)	Y	Y	?	N	?	Y	Y	Y	Y	Y	Y	Y	N	N	Poor
Duan et al. (2021)	N	N	N	N	?	Y	N	Y	?	Y	Y	?	N	N	Poor
Fekete et al. (2022)	Y	?	?	?	?	Y	N	Y	Y	Y	Y	Y	N	N	Poor
Frein et al. (2014): S1	Y	?	?	?	?	?	Y	Y	?	Y	Y	?	N	Y	Fair
Fuju et al. (2022)	Y	Y	?	N	?	Y	N	Y	?	Y	Y	N	Y	N	Poor
Gallagher et al. (2020)	Y	Y	Y	Y	?	Y	N	N	N	Y	Y	N	N	Y	Poor
Guastella et al. (2008)	Y	?	?	?	?	Y	Y	?	?	Y	Y	?	N	N	Poor
Hansen et al. (2021)	Y	Y	Y	N	?	Y	N	Y	Y	Y	Y	Y	Y	N	Poor
Heekerens et al. (2020)	Y	?	?	?	?	N	Y	Y	N	Y	N	Y	N	Y	Poor
Heekerens et al. (2022)	Y	Y	Y	?	?	Y	Y	Y	Y	Y	Y	N	N	Y	Fair
King et al. (2000)	Y	?	?	Y	?	?	Y	Y	Y	Y	Y	?	N	Y	Fair
King (2001)	Y	?	?	?	?	?	Y	Y	Y	Y	Y	?	N	Y	Fair
Kloss et al. (2002)	Y	Y	N	?	?	Y	Y	?	Y	Y	Y	?	N	?	Fair
Kupeli et al. (2018)	N	N	N	N	?	Y	N	Y	Y	Y	Y	?	N	Y	Poor
Layous et al. (2013)	Y	?	?	N	?	Y	Y	?	?	Y	Y	?	N	N	Poor
Layous et al. (2017) S3	Y	?	?	?	?	?	Y	Y	?	Y	N	Y	N	N	Poor
Lovell et al. (2016)	Y	Y	?	?	?	Y	Y	?	Y	Y	Y	N	N	N	Poor
Marlo et al. (1999)	Y	?	?	?	?	?	Y	?	?	Y	Y	?	N	Y	Fair
Moieni et al. (2019)	Y	?	?	?	?	Y	Y	Y	Y	Y	Y	N	N	N	Poor
Nagurney (2013)	Y	Y	Y	?	?	?	Y	Y	?	Y	Y	?	N	Y	Fair
Nelson et al. (2010)	Y	?	?	?	?	?	Y	Y	?	Y	Y	?	N	Y	Fair
Regan et al. (2023)	Y	?	?	?	?	?	N	Y	?	Y	Y	?	N	Y	Fair
Renner et al. (2014)	Y	?	?	?	?	Y	Y	Y	Y	Y	Y	?	N	Y	Fair
Round et al. (2022)	Y	?	?	N	?	Y	N	?	Y	Y	Y	Y	Y	N	Poor
Shapira et al. (2010)	Y	Y	Y	?	?	Y	N	?	?	Y	Y	?	N	Y	Poor
Shin et al. (2020)	Y	?	?	?	?	Y	Y	Y	?	Y	Y	Y	N	N	Poor
Smith et al. (2018)	Y	?	?	?	?	Y	Y	Y	Y	Y	Y	Y	N	N	Poor
Teismann et al. (2014)	Y	Y	N	?	?	Y	Y	Y	?	Y	Y	N	N	N	Poor
Timmons et al. (2018)	Y	?	?	N	?	Y	N	N	Y	Y	Y	N	N	N	Poor
Toepfer et al. (2009)	N	N	N	?	?	?	Y	Y	?	Y	Y	?	N	Y	Poor
Toepfer et al. (2012)	Y	?	?	N	?	?	Y	Y	?	Y	Y	?	N	Y	Fair
Toepfer et al. (2016)	Y	?	?	?	?	Y	Y	Y	?	Y	Y	?	N	Y	Fair
Troop et al. (2013)	Y	?	?	?	?	Y	Y	Y	?	Y	Y	?	N	Y	Fair
Walsh et al. (2022) S1	Y	?	?	?	?	?	Y	Y	?	Y	Y	Y	N	Y	Fair
Walsh et al. (2023)	Y	?	?	?	?	?	Y	Y	?	Y	Y	Y	Y	N	Poor
Wing et al. (2006)	Y	Y	Y	?	?	Y	Y	Y	Y	Y	Y	?	N	N	Poor
Wong et al. (2009)	Y	?	?	Y	?	?	Y	?	Y	Y	Y	?	N	N	Poor

### Study outcomes

Studies were divided based on the type of positive writing interventions and were discussed with respect to the following topic categories: best possible self, positive experiences, gratitude, benefit finding, satisfaction processes, three good things and resource diary. Main effects and interactions on outcomes are considered here and moderators are reported separately.

#### Best possible self (BPS).

Studies that described the intervention topic as writing about one’s best possible self (BPS) in various domains, an optimistic future, or life goals were considered BPS interventions. Eighteen studies employed this topic and the majority of studies (*n *= 11) used instructions based on the original BPS writing study conducted by King [8]. Most BPS interventions focused broadly on the future [8,22–26] and four studies included a post-writing imagery element [27–30]. Other studies altered the domain of self each day (e.g., academic, social, career or health) [31,32] assessed various time orientations (e.g., future, present, past) [33], focussed on romantic relationships [34,35] or focussed on the work-related self [36,37]. BPS topic did not seem to influence intervention effectiveness. In addition to heterogeneous BPS topics, there were considerable variations in the protocols employed and outcomes assessed. Outcomes were primarily psychological and only one study included a physical health outcome. Only one study recruiting undergraduate students found no health benefits following BPS in that subjective wellbeing outcomes improved in the control relative to BPS [37]. However, authors suggest that this might be due to conducting the study during the Covid-19 pandemic and the potential difficulties of writing about a future orientated task (BPS) relative to a past orientated task (neutral writing). Of the remaining BPS interventions, findings on psychological outcomes showed enhanced optimism [23,30,33], happiness [24,33], flourishing [31], flow [32], psychological wellbeing [8], self-efficacy and self-satisfaction [33], as well as reductions in ruminative thinking [25], psychological distress [35] and self-criticism [26]. For need satisfaction, benefits were only evident for autonomy [31] and relatedness facets [32], but not competence. Findings on positive affect, negative affect, depression and life satisfaction were mixed, though most studies found significant benefits across the measures. Eight studies found increases in positive affect [8,28–34] but three found no improvements at follow-up [23,25,26]. Negative affect decreased in five studies [29,30,33,34], but this decrease was not observed in two studies relative to controls [22,36]. Depression reduced in two studies [24,31], but another two observed no changes relative to controls [22,36]. Life satisfaction improved in two studies [33] but not in one [28]. No improvements were found for gratitude, hope [28], personal growth, restrictive emotionality [35] and stress [26]. In terms of physical health outcomes, reductions in health centre visits were found at a 5-month follow-up [8].

#### Positive experiences.

Writing about positive experiences included writing about intensely positive experiences (IPE), happy moments or any topic that encouraged individuals to reflect upon a positive memory or aspect of life. Sixteen studies implemented this intervention topic and the majority used instructions based on a study conducted by Burton and King [38] which involved writing about IPEs for 15–20 minutes once per day over three to four consecutive days. Seven studies followed this exact protocol [39–44]. Only one study employed two IPE interventions where one group had the addition of an emotional cue where participants were instructed to write about how they could tap into or recreate such inspiring feelings [45]. Two studies based their instructions on IPEs but altered the duration and spacing of sessions to two minutes per day over consecutive days [46] and four 20-minute sessions twice per week [47]. Two studies followed the same protocol as Burton and King's [38] IPE intervention, but used different instructions focussing broadly on positive and happiest experiences [48,49]. Other studies used more specific topics including writing about past positive experiences written in the present tense with focus on sensory details [50], focusing on happiest romantic relationships [51] and a past peak experience coping with a challenge [52]. These studies included primarily psychological health outcomes but assessed more physical health outcomes relative to the other topics. Four studies found no significant health benefits overall on outcomes including depression, trait anxiety [39,40,51], state anxiety, perceived stress, physical symptoms [39], affect [39,50] and post-traumatic cognitions [50]. For the remaining twelve studies, significant improvements in psychological wellbeing outcomes were found for optimism, stress appraisal [52], trait anxiety [44,49], positive affect [38,41,52], disordered eating [42], psychological health [47] and suicidal ideation [48]. Life satisfaction was also found to increase in IPE with the addition of an emotional cue; however this effect was not evident in the IPE group without the cue [45]. Findings on state anxiety were mixed where reductions were found in three studies [43,44,48] but two studies revealed no significant reductions [47,49]. Findings on negative affect, perceived stress and depression were also mixed where each improved in one study out of two studies. Some studies found reductions in negative affect [41,52], depression [48] and perceived stress [44], but some did not find any changes in negative affect [38], depression [42] and perceived stress [42,43]. Aspects of job satisfaction were improved in one study [43]. No improvements were found for burnout [43] and self-criticism/reassurance [42]. Findings on physical health outcomes were mixed. Self-reported physical symptoms were found to decrease in two studies [38,46] but did not improve in another three [39,47,49]. The number of health centre visits decreased in one study [38], but these effects were not evident in another two studies [48,49]. One study found that physical symptoms increased relative to controls following the positive writing intervention [47].

#### Gratitude letter.

Eleven studies assessed gratitude interventions which consisted of writing about aspects of life for which one is grateful [53,54] or a letter of gratitude towards a person of choice [55–57], parent(s) [58], or various recipients [59]. Four studies assessed multiple gratitude interventions comprising a generic gratitude letter towards a person of choice and child-specific letter towards their child with autism spectrum disorder [60], a gratitude letter and non-social letter [61], a kindness gratitude letter, health focussed gratitude letter and a work focussed gratitude letter [62] and a private gratitude letter, social media post and text message [63]. All outcomes were measured within a one-month follow-up period and only one study included a physical outcome measure. Significant improvements were found for elevation [62,63], happiness, life satisfaction [56,57,60,61,63], humility, improvement motivation, indebtedness [62], benefit finding, parenting self-efficacy, and optimism [60] and stress [53]. In terms of needs satisfaction, improvements were observed for autonomy and connectedness facets but not competence [61]. Findings on depression, gratitude and positive affect and negative affect were mixed. All but one study [60] assessed gratitude as an outcome and only two found no significant improvements [57,58]. Positive affect increased in four studies [54,58,61,63] but not one [53]. Reductions on negative affect were found in three studies [53,54,63] but not two [61,62]. Reductions were observed for depression in two studies [57,60] but not another [53]. No significant improvements were found for anxiety [53].

#### Benefit finding.

Benefit finding was described as interventions where participants were encouraged to write about the benefits or positives following a stressful or upsetting experience. Five studies employed this technique with a focus on either the caregiving experience [64,65], a personally identified upsetting or traumatic experience [7,66] and the Covid-19 pandemic [67]. Outcomes were primarily psychological, and few significant benefits were observed. Significant improvements were found for state anxiety immediately post-writing [67] and post-traumatic growth at a 2-month follow-up [66] relative to neutral writing controls. Findings on trait anxiety and depression were mixed. Two studies found decreases in trait anxiety however this was no different to controls in one study [67] and was relative to an increase in anxiety in the control group 3 months post-writing in another study [65] suggesting a buffering effect of the benefit finding intervention. Two other studies found no significant differences in trait anxiety [66,68]. Reductions in depression were only observed in one study [67]; however this was also no different to controls and three other studies found no significant improvements [64–66]. No improvements were found for affect [7], benefit finding, caregiver quality of life [64], or perceived stress [54,67,69]. In terms of physical health outcomes, one study observed fewer health centre visits following benefit finding writing relative to controls [7]. No significant improvements were found for physical symptoms [67].

#### Satisfaction processes.

One study investigated the effects of writing about satisfaction processes and associated positive emotions derived from engaging meaningful experiences, focusing on aspects such as pleasure and enjoyment, sense of involvement, potential for development, and losing track of time [70]. This was completed three times for 20 minutes, once per day over consecutive days. Improvements were found for all psychological outcomes including life satisfaction, positive affect, psychological wellbeing, social wellbeing, depression, anxiety and stress at a two-week follow-up when compared to a neutral writing control. No benefits were found for self-reported general physical health

#### Three good things.

One study employed a three good things intervention which involved writing three good things that happened at the end of each day with reasons, daily for four weeks [71]. Improvements were found in depression and positive cognitive appraisal at the end of the intervention relative to a daily food diary control. No improvements were observed for caregiver burden, caregiver distress, positive feelings and quality of life.

#### Resource diary.

One study assessed the use of a resource diary intervention which involved writing about inter- and intra-personal resources three times per week for 12 weeks [72]. No significant main effects were found on resource realisation outcomes immediately post-treatment.

### Moderators

Fifteen moderators were identified in the review. In two BPS interventions, low emotional processing coping, and low emotional expressive coping was associated with decreases in depression [36], hostility and negative affect [22], and both studies showed that these traits led to fewer health centre visits. However, one BPS study found no significant moderation for emotional self-awareness on positive wellbeing outcomes [23]. One BPS study found that low dispositional pain catastrophising was associated with greater reductions in situational pain catastrophising following thermal stimulation, however no moderation was found for dispositional optimism [27]. For IPE interventions, greater reductions in depression and trait anxiety were observed for those with low alexithymia [40] and greater reductions in depression and perceived stress reactivity were found for those with high social inhibition [39], but no moderation was found for Type D personality [44]. Within gratitude letter interventions, greater benefits were found with gratitude and positive affect for those with high perceived familial collectivism and poorer perceived parent-child relationship [58]. Psychological distress [59], race [58] and trait gratitude [23] were not found to moderate gratitude intervention effectiveness. Greater benefits were found in resource realisation following a resource diary intervention for those with lower baseline wellbeing and lower baseline brooding [72]. One benefit finding study assessed perseverative thinking as a moderator, but this was non-significant [67].

## Discussion

This review synthesised 51 studies assessing the effects of positive expressive writing interventions on physical and psychological wellbeing in non-clinical populations in order to determine the optimal conditions under which health benefits occur. Both within and between the seven types of interventions identified there was substantial heterogeneity in writing topics, methodologies implemented, and health outcomes assessed and affected. Psychological wellbeing and subjective wellbeing outcomes showed the most consistent benefits, however findings on psychological health and physical health outcomes were mixed. Across the different types of positive writing techniques in terms of psychological health and wellbeing, gratitude letter and BPS interventions revealed the most promising findings whereby health outcomes either showed consistent improvements across all studies, or where findings were mixed, the majority revealed significant benefits. On the other hand, positive experiences interventions revealed more mixed findings and benefit finding interventions revealed very few significant beneficial effects. Satisfaction processes and three good things revealed mixed findings and no benefits were found for a resource diary intervention. However, satisfaction processes, three good things and resource diary activities were each only assessed in one study. Therefore, drawing conclusions on the efficacy of these interventions is limited and warrants further investigation.

At a glance, it could appear that BPS and gratitude writing interventions are most effective in improving psychological health and wellbeing relative to positive experiences and benefit finding interventions, however one potential interpretation for differences in intervention efficacy between topics could be due to the chosen health outcomes. BPS and gratitude interventions primarily included psychological outcomes that measure aspects of wellbeing related to positive emotion or cognitive evaluations (e.g., happiness, life satisfaction, optimism). On the other hand, positive experiences and benefit finding interventions assessed a larger number of psychological health outcomes (e.g., anxiety, stress, depression). Although PPIs serve the purpose of reducing and mitigating against negative health symptoms, they primarily focus on cultivating and sustaining positive emotions and promoting positive wellbeing [4]. This is further supported in the current review where studies assessed immediate changes in affect from pre- to post-writing, and a greater number of studies showed increases in positive affect, but fewer studies showed decreases in negative affect. These findings are consistent with previous reviews on gratitude interventions [14,15] and BPS writing interventions [18] whereby greater improvements tend to be observed for positive emotion and subjective wellbeing outcomes, whereas smaller effects emerge for negative emotion and psychological health outcomes. In terms of physical health, there are mixed findings for all intervention topics which is also supported by previous reviews on gratitude interventions [16,17]. Furthermore, the Frattaroli [2] meta-analysis on experimental disclosure studies found that physical health outcomes improved more in populations with a pre-existing health condition, therefore the lack of findings in the current review could potentially be due to ceiling effects resulting from refining the current review to non-clinical samples.

Regarding whom positive writing interventions work for, the majority of the studies in the review were conducted in student populations. Although findings are mixed regarding intervention efficacy, this could indicate that student populations could benefit from positive writing interventions, and evidence suggests that positive psychology interventions in particular are well received in education, fostering student health, relationships, happiness and academic success [73]. In addition, there were a number of studies that recruited from a caregiver population who are in particular need of a low-intensity intervention [6]. Benefits were mostly observed for gratitude writing [60], but less so for positive experiences [40] and benefit finding interventions [64,65]. However, it is worth noting the aforementioned discussion regarding differences in outcomes assessed between these writing topics. In addition, several studies assessed individual differences as moderators of interventions effectiveness, although it is difficult to draw robust conclusions here because each moderator was only assessed in a single study. Findings showed that individual differences relating to various emotional, social and wellbeing factors moderated intervention effectiveness. Therefore, future researchers should aim to replicate these moderation effects to determine their robustness.

With regards to how the intervention should be delivered, there was great heterogeneity in intervention delivery which makes it challenging to draw inferences regarding intervention effectiveness based on factors such as the writing protocol, control group, the writing duration and length of follow-up. There were some consistencies observed whereby significant health benefits tended to occur within a 1-month follow-up period which is consistent with previous reviews on emotional disclosure studies [2]. No clear differences were observed on the basis of number, duration or spacing of sessions. Moreover, there were no clear differences in findings based on the type of control group employed. The review also highlights that these interventions could be effective regardless of whether they are administered by typing or handwriting, which supports the use of positive writing as an accessible intervention that can reach a wide range of individuals.

The quality assessment showed that many studies were rated as poor due to not analysing all randomised participants using appropriate procedures. It has been previously recognised that few expressive writing studies include intention-to-treat analyses, and inclusion of this variable on a quality rating scale may only have limited utility [2]. However, it is imperative that future researchers adopt intention-to-treat or other methods of analyses where all randomised participants are included, as completers only analyses are susceptible to attrition bias resulting from differential dropout rates. This could potentially threaten the internal validity of the studies and make it difficult to draw conclusions regarding treatment effects. However, the quality assessment showed that most studies had low differential dropout which could indicate that there are other factors influencing retention, and it is not due to dissatisfaction with the specific condition participants are allocated to. Future researchers should aim to employ more rigorous methods and reporting protocols, such as following the CONSORT checklist for randomised controlled trials [74].

Although the review contributes to the ongoing assessment of positive expressive writing interventions, it is worth noting several limitations from the review. Firstly, the purpose of positive psychology interventions is not only to reduce or prevent the worsening of psychopathological symptoms (e.g., stress, anxiety, depression), but also to facilitate and enhance positive wellbeing. Therefore, it would have been appropriate to include a broader range of search terms relating to subjective wellbeing outcomes (e.g., positive affect, happiness, flourishing). In addition, the quality assessment tool used in this review has not been independently published and is not considered standardised. It could be argued that the Cochrane Collaboration’s tool [75] would be a more suitable and rigorous method of quality assessment, however, the NHLBI includes criteria that assess the key domains of selection bias, performance bias, detection bias and attrition bias which are essential to draw valid conclusions about the effectiveness of an intervention or treatment. With these criteria in mind, only issues regarding selection bias and attrition bias were identified, and the majority of studies in the review were low quality despite using a less rigorous tool, therefore this critique may carry relatively less substantial significance. A final limitation is that the studies in the review were largely conducted in western and university populations. PPIs have previously been critiqued for largely being a Western, Educated, Industrialized, Rich and Democratic (WEIRD) enterprise [76], which limits current understanding of the efficacy of such interventions across different cultures and underrepresented groups. Therefore researchers should aim to assess positive writing in non-WEIRD populations in future.

In conclusion, this review highlights the heterogeneity of health and wellbeing outcomes assessed and methods implemented which limits the ability to find consistencies in findings. There is indication that positive writing more reliability enhances wellbeing and positive affective states, whereas findings are mixed regarding the effects on negative affect, psychological health, and physical health outcomes. BPS and gratitude writing interventions appear to show the greatest health benefits however it is uncertain as to whether this is due to the positive topic, or the larger number of wellbeing outcomes assessed. In addition, this review highlights that individual differences relating to emotional, social and wellbeing factors may moderate intervention effectiveness. Future researchers should include both health and wellbeing outcomes and include individual differences as moderators when investigating the effectiveness of positive writing and more rigorous methods should be employed.

## Supporting information

S1 File PRISMA 2020 checklist. (PDF)

S2 File Data extraction. (XLSX)

S3 File Full-text screening. (XLSX)

S4 File Quality assessment. (XLSX)
